# Coronary slow flow and its correlation with reduced left ventricle global longitudinal strain: a case–control study

**DOI:** 10.1186/s44156-023-00037-6

**Published:** 2024-01-10

**Authors:** Ahmed Shawky Shereef, Mohamed Gamal Abdelmajeed, Mohamad Hossam Alshair, Ibtesam Ibrahim El-Dosouky, Wael Ali Khalil, Shaimaa Wageeh, Islam Elsayed Shehata

**Affiliations:** Department of Cardiology, Faculty of Medicine, Zagazig University, Zagazig, 44519 Egypt

**Keywords:** Coronary angiography, Coronary slow flow, Echocardiography, Myocardial dysfunction, LV strain

## Abstract

**Background:**

Coronary slow flow (CSF) often links to inflammation and endothelial function disturbance. While conventional ejection fraction measurements fall short in identifying myocardial dysfunction, left ventricular global longitudinal strain (LV GLS) has shown superior efficacy in this regard. Our study aimed to explore subclinical left ventricular systolic dysfunction by assessing LV GLS in patients diagnosed with coronary slow flow (CSF).

**Methods:**

The study included sixty patients with CSF and sixty control individuals without CSF. Coronary angiography employed the Thrombolysis in Myocardial Infarction (TIMI) frame count (TFC) to identify CSF. LV GLS values were evaluated and compared between the two groups.

**Results:**

Significantly reduced LV GLS was evident in the CSF group compared to the control group (− 16.18 ± 1.25 vs. − 19.34 ± 1.33, *p* < 0.001). A notable correlation (r = 0.492, *p* < 0.001) between LV GLS and TFC was observed in the CSF group. Multivariate logistic regression analysis highlighted reduced LV-GLS (OR 2.2, 95% CI 1.57–3.09, *p* < 0.001) and smoking (OR 11.55, 95% CI 3.24–41.2, *p* < 0.001) as significant predictors for CSF presence. The receiver operating characteristic curve established that an LV GLS value of ≥ − 17.8% accurately predicted the presence of CSF (AUC: 0.958, 95% CI: 0.924–0.991, *p* < 0.001) with 90% specificity and 91.7% sensitivity.

**Conclusion:**

Our study indicates that reduced LV GLS is associated with CSF presence, offering a valuable means to early detect subclinical left ventricular systolic dysfunction in high-risk patients susceptible to heart failure.

*Trial registration:* ZU-IRB#7038/12-7-2021 Registered 12 July 2021, email: IRB_123@medicine.zu.edu.eg.

## Background

Coronary slow flow (CSF) constitutes a significant clinical concern characterized by delayed opacification of non-obstructed coronary vessels [1]. Among patients undergoing coronary angiography for stable angina pectoris, the incidence of CSF ranges from 1 to 7%. Notably, over 80% of these patients experience recurrent chest discomfort, while nearly 20% present with clinically significant symptoms requiring readmission [2].

The etiological factors underlying CSF involve microvascular dysfunction, endothelial dysfunction, and inflammation [3]. Despite these identified factors, the precise pathophysiological mechanisms driving this distinctive angiographic phenomenon and its clinical implications remain inadequately understood. In individuals with ostensibly normal coronary arteries, CSF may induce transient myocardial hypoperfusion, elevating the risk of coronary artery disease (CAD) and exacerbating the overall prognosis [4]. The influence of endothelial and microvascular dysfunction on cardiac systolic function in patients with CSF remains an area devoid of comprehensive understanding.

Left ventricular ejection fraction (LVEF) fails to accurately depict intrinsic myocardial contractility, often appearing normal despite reduced LV systolic function [5]. The assessment of LVEF is hampered by substantial intra- and inter-observer variability and significant dependency on loading conditions [6]. Evaluation of myocardial strain presents a potential workaround for several limitations associated with LVEF in appraising LV systolic function. Utilizing speckle tracking echocardiography, myocardial strain can be assessed in three spatial directions (longitudinal, radial, and circumferential), irrespective of the ultrasound beam's angle of incidence. Longitudinal strain is a commonly employed metric in clinical settings to gauge LV systolic function and is considered superior to radial and circumferential strains in assessing systolic function [7]. Left ventricular global longitudinal strain (LV-GLS) serves as a reliable indicator of cardiac dysfunction, holding substantial long-term prognostic relevance across various cardiac conditions [8]. Some studies have suggested that LV-GLS, as a noninvasive marker, exhibits a strong correlation with CAD severity, and an aberrant LV GLS value could potentially identify myocardial damage before a decline in LV ejection fraction (EF) occurs [9, 10]. In our investigation, we specifically studied LV GLS, assessed through 2D speckle tracking, as a sensitive marker of subclinical left ventricular dysfunction in patients diagnosed with CSF.

## Methods

### Ethical considerations

The study protocol received approval from the University Institutional Review Board (IRB) and was conducted in compliance with the 1975 Declaration of Helsinki, as confirmed by the institution's human research committee's prior endorsement. Written informed consent was obtained from all participating individuals.

### Study design and study population

This case–control investigation was conducted at the Cardiology Department of our University Hospitals between August 2021 and May 2023. The study encompassed sixty patients diagnosed with CSF and an equivalent number of sixty control subjects with normal coronary arteries and no CSF. Verification of CSF was performed through coronary angiography, utilizing the Thrombolysis in Myocardial Infarction (TIMI) frame count (TFC) to assess coronary blood flow velocity. All participants underwent coronary catheterization due to indications of myocardial ischemia from non-invasive tests, typical angina not responsive to medical intervention during minimal activity, typical angina presenting high-risk events in clinical evaluations, or a high clinical probability of coronary artery disease (CAD) without prior non-invasive risk stratification [11]. Patients with a history of cardiac surgery, percutaneous coronary intervention (PCI), or diagnosed with CAD, including those with spasm, myocardial bridge, coronary aneurysm, coronary ectasia, obstructive lesions, or myocardial infarction, were excluded from the study. Additionally, individuals with congenital heart diseases, left ventricular hypertrophy, chronic diseases, hematological disorders, valvular diseases, an ejection fraction below 50%, abnormal cardiac rhythms, or conduction abnormalities were excluded.

### Clinical assessment

A comprehensive and detailed medical history was obtained, encompassing risk factors associated with coronary artery disease (CAD), along with a record of any anti-ischemic medications previously used. Hypertension was defined as a blood pressure reading equal to or exceeding 140 mmHg systolic or 90 mmHg diastolic, and individuals undergoing anti-hypertensive therapy were also classified as hypertensive. Diabetes mellitus was determined by an HbA1C level greater than 6.5 g/dL or the use of anti-diabetic medications. Active smoking within the preceding six months was considered as a determinant for the classification of smoking. Dyslipidemia was defined by serum cholesterol levels above 200 mg/dL, HDL levels below 40 mg/dL, LDL levels above 130 mg/dL, or the use of anti-dyslipidemic medications. A family history of premature CAD was ascertained if a first-degree relative experienced a cardiovascular event before the age of 55 for males and 65 for females. Body Mass Index (BMI) was calculated using the ratio of weight in kilograms to the square of height in meters. Blood pressure assessments, heart rate evaluations, and electrocardiograms (ECG) were conducted for all participants.

### Invasive coronary angiography

During invasive coronary angiography conducted in our catheterization laboratory (Philips Integris 5000, Netherlands), six French diagnostic catheters—specifically Judkins right and left—were utilized to access the right and left coronary arteries. For contrast enhancement, an ionic contrast agent (ioxitalamic acid; Telebrix-35, 350 mg/ml) was manually injected at a volume ranging from six to ten ml at each position. Intracoronary injections of 100–200 µg of nitroglycerine were administered to all patients.

Employing the Thrombolysis in Myocardial Infarction (TIMI) frame count (TFC) method, established by Gibson et al. [12], two cardiologists, blinded to the patients' clinical information, evaluated coronary artery flow. This strategy involves measuring cine frames by calculating the difference between the first and distal frames, typically viewed at a rate of 30 frames per second. Distal landmarks were pre-defined for each major coronary artery: the left anterior descending artery's (LAD) distal bifurcation (referred to as the "whale's tail" or "pitchfork") for the LAD, the distal bifurcation of the segment with the longest total distance for the left circumflex (LCX), and the first branch of the posterolateral artery for the right coronary artery (RCA). Given the LAD's usual greater length compared to other coronary arteries, the TFC of the LAD was divided by 1.7 to obtain the corrected TFC (CTFC). The mean CTFC was calculated by averaging the CTFCs obtained from all three vessels. The criteria for diagnosing coronary slow flow were based on guidelines set forth by Beltrame JF [13], which necessitated at least one main coronary artery, viewed at 30 frames per second, exhibiting a CTFC value exceeding 27 frames, with no significant stenosis or stenosis less than 40% observed during coronary angiography.

### Conventional transthoracic echocardiography

Using the M5Sc-D probe and the Vivid E95 system (GE Healthcare Ultrasound, Horten, Norway), a comprehensive 2D transthoracic echocardiogram was conducted within 48 h of the coronary angiography. Image acquisition was performed in the left lateral position at the end of expiration, adhering to the data acquisition guidelines recommended by the American Society of Echocardiography [14], which included 2D, Doppler (continuous wave, pulsed wave, and color Doppler), and Tissue Doppler Imaging (TDI) modalities. Ejection fraction was estimated using the Biplane Simpson's approach.

The presence of left ventricular diastolic dysfunction (LVDD) was determined if more than two of the following criteria were met: (1) Average E/ e > 14; (2) Septal e velocity < 7 cm/s or lateral e` velocity < 10 cm/s; (3) Tricuspid regurgitation (TR) velocity > 2.8 m/s; (4) Left atrial volume index > 34 mL/m^2^ [15].

### 2D speckle tracking study

A 2D speckle tracking analysis was conducted utilizing the same imaging device and probe. Grey scale images capturing three cardiac cycles (at a frame rate of 60–80 frames/s) were obtained from apical views of two, three, and four chambers. Initially, the left ventricle's endocardial border was automatically defined, followed by manual adjustments, if necessary, to delineate both the epicardial and endocardial borders accurately. The region of interest widths were adjusted to ensure proper visualization of the real epicardial and endocardial borders. The estimation of left ventricular global longitudinal strain (LV-GLS) was derived by computing the average of segmental strain values obtained from all left ventricular segments across all imaging views.

### Statistical methods

The statistical analysis was conducted using SPSS version 20. The distribution model of quantitative data was evaluated utilizing the Kolmogorov-Smirnov test. For normally distributed data, the mean and standard deviation were calculated, and the independent sample t-test was employed. Qualitative data were analyzed using numbers and percentages along with the Chi-square test. In determining independent predictors of CSF, both univariate and multivariate regression analyses were carried out using the Enter method. The receiver operating characteristic (ROC) curve was utilized to establish the optimal cut-off values of LV-GLS for predicting CSF. Pearson's correlation coefficient was applied to assess the correlation between LV-GLS and other continuous variables in the CSF group. Statistical significance was defined as a *p*-value less than 0.05.

## Results

The study comprised 60 patients in the CSF group, consisting of 16 females and 44 males, with an average age of 44.58 ± 4.92 years. The control group included 60 individuals (33 females and 27 males) with an average age of 43.53 ± 5.01 years. A significantly higher proportion of males, hypertensive individuals, and smokers were observed in the CSF group (*p* < 0.05). Additionally, the CSF group exhibited a significantly higher BMI (*p* < 0.05) (Table 1).

**Table 1:** Baseline demographic and clinical characteristics of the participants

Variables	CSF group (n = 60) Mean ± SD Number (%)	Control group (n = 60) Mean ± SD Number (%)	*p-value*
Male gender	44(73.3)	27(45.0)	0.002*
Age (years)	44.58 ± 4.92	43.53 ± 5.01	0.249
Hypertension, n (%)	34 (56.7)	19 (31.7)	0.006*
Diabetes mellitus, n (%)	22 (36.7)	18 (30)	0.439
Smoking, n (%)	44 (73.3)	18 (30)	< 0.001*
Dyslipidemia, n (%)	31 (51.7)	21 (35)	0.65
Family history of premature CAD, n (%)	21 (35)	16 (26.7)	0.323
Medications			
Aspirin	36 (60)	33 (55)	0.58
Beta-blockers	24 (40)	20 (33.3)	0.44
ACEIs/ ARBs	15 (25)	13 (21.7)	0.67
Calcium channel blockers	12 (20)	9 (15)	0.42
Nitrates	24 (40)	27 (45)	0.58
Statins	27 (45)	32 (53.3)	0.52
BMI (kg/m^2^)	27.29 ± 2.63	25.7 ± 2.69	0.001*
Diastolic blood pressure (mmHg)	80.73 ± 2.46	80.62 ± 4.93	0.87
Systolic blood pressure (mmHg)	116.65 ± 12.96	119.67 ± 11.4	0.184
Heart rate (bpm)	80.38 ± 9.21	77.53 ± 7.599	0.067

Data are given in mean ± SD or number and frequency, *p < 0.05

*ACEIs* angiotensin-converting enzyme inhibitors, *ARBs* angiotensin receptor blockers, *BMI* body mass index, *CSF* coronary slow flow phenomenon, *SD* standard deviation

**Fig. 1:**
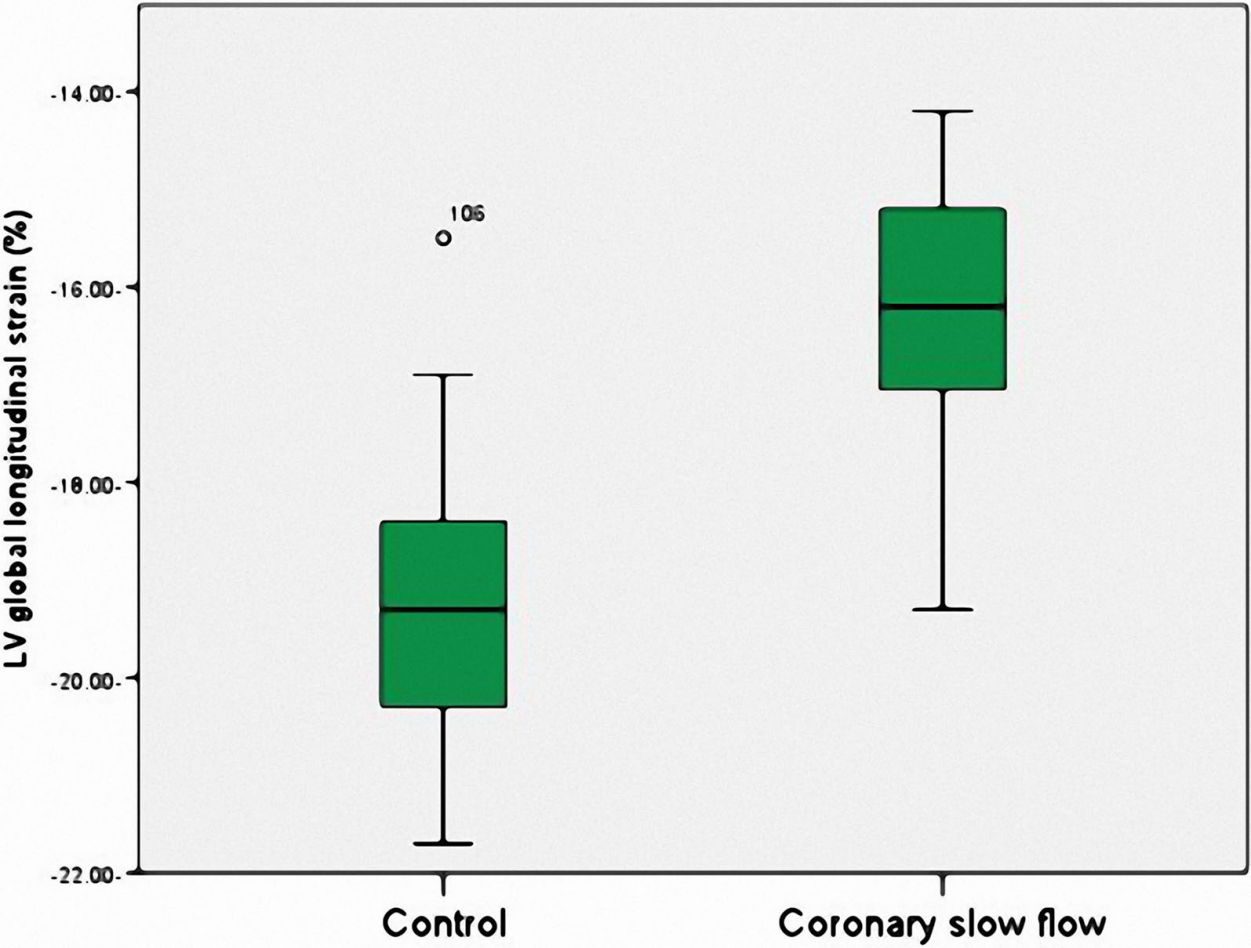
Comparison of left ventricle global longitudinal strain values in the coronary slow flow group and the control group

In Table 2 and Fig. 1, a significantly reduced global longitudinal strain (LV-GLS) was evident in the CSF group (*p* < 0.001). However, there were no substantial differences between the groups concerning other echocardiography findings (*p* < 0.05). Furthermore, the corrected thrombolysis in myocardial infarction frame counts (CTFCs) for the main coronary arteries were significantly higher in the CSF group (*p* < 0.001).

**Table 2:** Echocardiographic findings and TIMI frame count of the participants

Variables	CSF group (n = 60) Mean ± SD Number (%)	Control group (n = 60) Mean ± SD Number (%)	*p*
LVEF (%)	61.08 ± 5.27	61.48 ± 4.91	0.668
LVEDV (ml)	87.8 ± 5.28	88.47 ± 5.67	0.506
LVESV (ml)	34.15 ± 4.64	34.03 ± 4.31	0.887
E/A	0.78 ± 0.19	0.83 ± 0.17	0.079
Septal e′	6.92 ± 2.48	7.13 ± 1.49	0.563
Lateral e′	8.98 ± 2.05	9.33 ± 1.75	0.316
Average E/e`	8.85 ± 1.62	8.27 ± 1.69	0.056
Left atrial volume index	32.3 ± 4.89	31.62 ± 4.69	0.436
Tricuspid regurge velocity	2.08 ± 0.77	1.95 ± 0.79	0.35
LV diastolic dysfunction	29 (48.3)	20 (33.3)	0.095
LV global longitudinal strain (%)	− 16.18 ± 1.25	− 19.34 ± 1.33	< 0.001*
TIMI frame count			
LAD (corrected)	41.33 ± 5.18	17.99 ± 1.85	< 0.001*
LCX	39.03 ± 5.5	19.23 ± 2.76	< 0.001*
RCA	39.28 ± 6.58	20.58 ± 2.4	< 0.001*
Mean (corrected)	39.88 ± 3.94	19.27 ± 1.41	< 0.001*

Data are given in mean ± SD or median and range, *p < 0.05

*CSF* coronary slow flow, *SD* standard deviation, *TIMI* thrombolysis in myocardial infarction, *LCx* left circumflex coronary artery, *LAD* left anterior descending coronary artery, *RCA* right coronary artery, *LVEF* left ventricular ejection fraction, *LVEDV* left ventricular end-diastolic volume, *LVESV* left ventricular end-systolic volume, *E/A* ratio of early to late diastolic trans-mitral flow velocity, *E/e`* ratio of mitral to annular early diastolic peak velocity

**Fig. 2:**
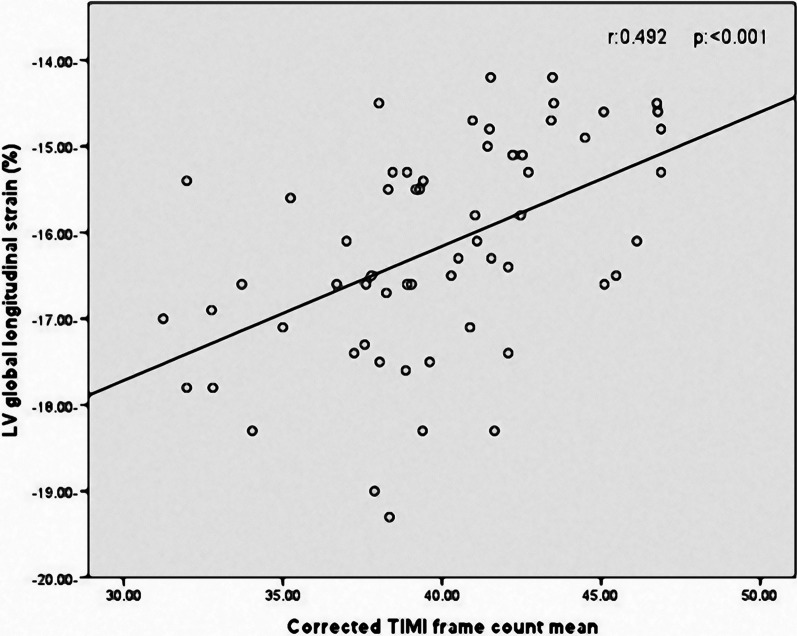
Correlation between LV-GLS and corrected TIMI frame count in patients with CSF

**Fig. 3:**
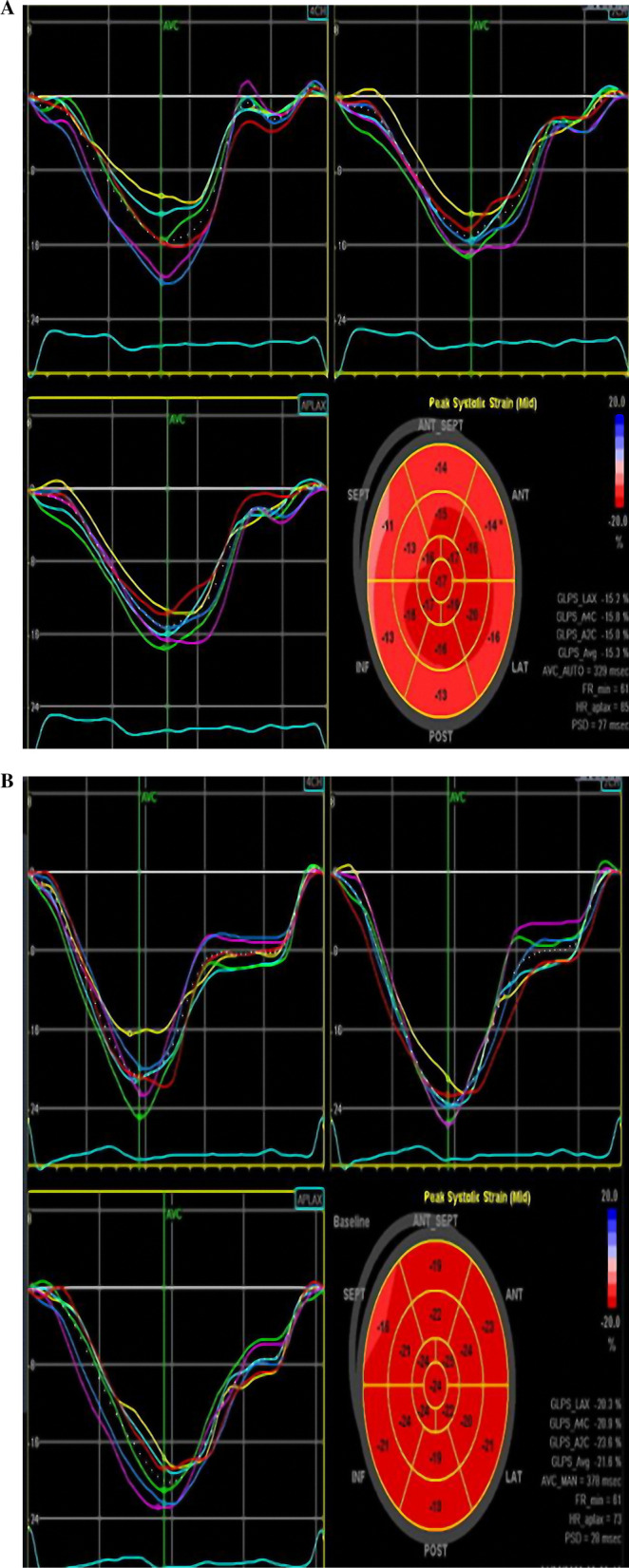
**A** Left ventricular global longitudinal strain in patients with coronary slow flow (GLS = − 15.3). **B** Left ventricular global longitudinal strain in patients without coronary slow flow (GLS = − 21.6)

Correlation analysis revealed that LV-GLS in patients with coronary slow flow was significantly associated with age (r = 0.259,* p* = 0.046), BMI (r = 0.257, *p* = 0.047), heart rate (r = 0.273, *p* = 0.035), and CTFC mean (r = 0.492, *p* < 0.001) (Fig. 2). Figure 3 illustrates two LV-GLS measurements, one for a patient with CSF (A) and the other for a control individual (B).

**Fig. 4:**
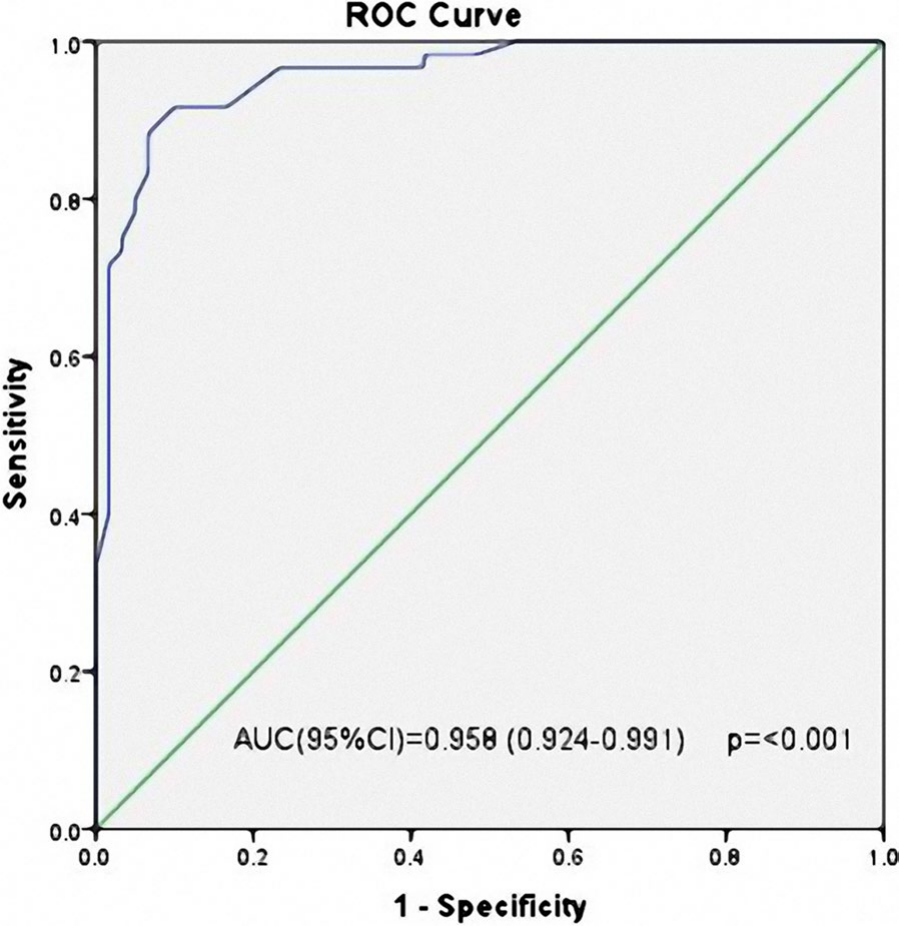
Receiver operating characteristic curve of left ventricle global longitudinal strain values for prediction of the presence of coronary slow flow

Hypertension, smoking, BMI, LV-GLS, left ventricular ejection fraction (LV EF), and left ventricular diastolic dysfunction were included in the multivariate analysis to predict the presence of CSF. Male gender was excluded due to collinearity with smoking. Multivariate regression analysis identified reduced LV-GLS (OR 2.2, 95% CI 1.57–3.09, *p* < 0.001) and smoking (OR 11.55, 95% CI 3.24–41.2, *p* < 0.001) as significant independent predictors of CSF (Table 3). The ROC curve determined that an LV-GLS ≥ − 17.8% accurately predicted the presence of CSF (AUC: 0.958, 95% CI: 0.924–0.991, *p* < 0.001) with a specificity of 90% and a sensitivity of 91.7% (Fig. 4).

**Table 3:** Univariate and multivariate logistic regression analysis of predictors for the presence of coronary slow flow

Predictors	Univariate				Multivariate			
	OR	95% C.I		*p*	OR	95% C.I		*p*
		Lower	Upper			Lower	Upper	
Male	3.36	1.56	7.22	0.002				
Hypertension	2.82	1.34	5.95	0.006	4.01	1.2	13.43	0.024
Smoking	6.42	2.9	14.21	< 0.001	11.55	3.24	41.2	< 0.001*
Body mass index	1.25	1.09	1.44	0.002	1.33	1.04	1.71	0.065
LV global longitudinal strain	6.29	3.21	12.32	< 0.001	2.2	1.57	3.09	< 0.001*
LV ejection fraction	0.98	0.92	1.06	0.665	1.04	0.93	1.16	0.484
LV diastolic dysfunction	0.53	0.26	1.12	0.096	2.42	0.75	7.79	0.14

*OR* odds ratio, *CI* confidence interval

^*^p < 0.05

## Discussion

The most important finding in our study is that LV-GLS was significantly associated with the presence of CSF. Moreover, the ROC curve of LV-GLS values demonstrated that the cut-off value has satisfactory specificity and sensitivity for the presence of CSF. Also, we found a significant correlation between LV GLS values and the CTFC mean.

CSF is a distinct angiographic finding and needs to be evaluated as a separate entity with distinctive characteristics, pathogenic pathways, and diagnostic features [1]. It can be complicated by a variety of clinical outcomes, including acute coronary syndrome, angina, heart failure, and sudden death [16]. It is unclear what causes this phenomenon until now. There are many ideas and speculations to explain its pathophysiology; however, microvascular and endothelial dysfunction are strongly suggested [13]. Also, it was thought in prior research to be the initial phase of atherosclerosis [17]. All these mechanisms contribute to a reduction in the coronary blood flow, leading to LV-GLS worsening.

The most commonly used tool in clinical practice to assess LV function is conventional echocardiography. However, this method is insensitive to mild myocardial dysfunction. GLS is a new common way to quantify ventricular function. It has been demonstrated to be a reliable measurement superior to the traditional ejection fraction, most likely as a result of the high degree of automation in the assessment and broad spatial averaging [18]. Compared to LVEF, LV-GLS plays a larger role in predicting cardiovascular outcomes [19]. Speckle-tracking echocardiography assesses both global and regional LV function. Peak global longitudinal strain (GLS) can measure systolic function and identify subclinical systolic dysfunction before LVEF declines, according to earlier research [20]**.** LV GLS has the ability to diagnose and rule out acute coronary heart disease more effectively than LVEF [21]. So, knowing the LV-GLS in patients with CSF is crucial to detecting high-risk patients for heart failure and other complications. We measured LV-GLS to evaluate the function of the LV in both groups. In our study, LV-GLS was significantly more impaired in CSF patients, although other traditional echocardiography parameters, including ejection fraction and diastolic function parameters were not significantly different between the two groups. This was in agreement with Wang et al. [22], who reached the same result concerning LV GLS among the two groups. Also, Altunkas et al. [23] discovered that traditional echocardiographic indices of systolic and diastolic function, such as LVEF, E/A and E/e' are inadequate at identifying subclinical systolic or diastolic impairment in CSF patients. ROC curve of LV-GLS values, demonstrating that the cut-off value has high specificity and sensitivity for the identification of CSF. This finding promotes LV-GLS as a non-invasive parameter to predict the presence of coronary slow flow. Our study revealed a significant association between the CTFC mean, which denotes to some extent the degree of ischemia, and LV-GLS. The myocardial longitudinal function significantly deteriorated as the mean TIMI frame count increased. Sucato et al. [24] reached the same result for those who had microvascular angina and ischemia but no obstructive coronary artery. This highlights the need to pay close attention to those with a higher TFC, as they have a higher risk of developing heart failure.

Additionally, in our study, smoking was proven to be significantly associated with the presence of CSF. Consistent with this finding, Ghaffari et al. [25] concluded a significant association between smoking and CSF, but Selcuk et al. [26] found that smoking was not significantly related to the presence of CSF. We can explain our finding by the fact that smoking is linked to oxidation, lower endogenous antioxidant concentrations, and a reduction in endothelium-dependent flow-mediated dilatation [27].

## Conclusions

This study underscores that subclinical left ventricular systolic dysfunction, as indicated by reduced LV-GLS values, is apparent in patients diagnosed with CSF and significantly correlated with CTFC. The application of LV-GLS as a precise measurement holds promise for risk stratification among patients with CSF, offering the potential to identify individuals at risk of developing heart failure.

### Limitations and recommendations:

Several limitations were identified in this study. First, the research was conducted with a limited sample size in a singular center, potentially limiting the generalizability of the findings. Second, the sole reliance on visual assessment of coronary arteries introduces operator-dependent bias. Employing intravascular imaging techniques like intravascular ultrasonography (IVUS) is recommended to enhance the accuracy of diagnostic results in assessing coronary artery diseases.

Additionally, the absence of follow-up in this study precludes the understanding of the longitudinal progression and outcomes in patients diagnosed with CSF. Future investigations should endeavor to address these limitations, incorporating larger sample sizes, multi-center approaches, and more comprehensive diagnostic methodologies to validate and extend the findings presented in this study. This would enable a more thorough understanding of the implications of LV-GLS in predicting adverse outcomes and the development of heart failure in individuals with CSF.

## Data Availability

Our data used to support the findings of this study are available from the corresponding author upon request.

## Abbreviations

BMI: Body mass index
CAD: Coronary artery disease
CBC: Complete blood count
CSF: Coronary slow flow
CSFP: Coronary slow flow phenomena
CTFC: Corrected Thrombolysis in Myocardial Infarction frame count
DBP: Diastolic blood pressure
ECG: Electrocardiogram
eNOS: Endothelial NO synthase
HDL: High-density lipoprotein
IVUS: Intravascular ultrasonography
LAD: Left anterior descending artery
LCX: Left circumflex
LDL: Low-density lipoprotein
LV GLS: Left ventricular global longitudinal strain.
PCI: Percutaneous coronary intervention
RCA: Right coronary artery
ROC: Receiver operating characteristic
SBP: Systolic blood pressure
TFC: TIMI frame count
TIMI: Thrombolysis in myocardial infarction
ZU-IRB: Zagazig University-Institutional Review Board
